# Eight Personal Characteristics Associated with the Power to Live with Disasters as Indicated by Survivors of the 2011 Great East Japan Earthquake Disaster

**DOI:** 10.1371/journal.pone.0130349

**Published:** 2015-07-01

**Authors:** Motoaki Sugiura, Shosuke Sato, Rui Nouchi, Akio Honda, Tsuneyuki Abe, Toshiaki Muramoto, Fumihiko Imamura

**Affiliations:** 1 International Research Institute of Disaster Science, Tohoku University, Sendai, Japan; 2 Institute of Development, Aging and Cancer, Tohoku University, Sendai, Japan; 3 Frontier Research Institute for Interdisciplinary Sciences, Tohoku University, Sendai, Japan; 4 Faculty of Humanities, Yamanashi Eiwa College, Kofu, Japan; 5 Graduate School of Arts and Letters, Tohoku University, Sendai, Japan; Southwest University, CHINA

## Abstract

People perceive, judge, and behave differently in disasters and in a wide range of other difficult situations depending on their personal characteristics. The power to live, as captured by characteristics that are advantageous for survival in such situations, has thus far been modeled in arbitrary ways. Conceptualizing such characteristics in more objective ways may be helpful for systematic preparations for future disasters and life difficulties. Here, we attempted to identify the major factors of the power to live by summarizing the opinions of survivors of the 2011 Great East Japan Earthquake disaster. We conducted personal interviews with 78 survivors about their survival experiences and elicited their opinions about the power to live as relevant to those experiences. We then incorporated these opinions into a questionnaire that was completed by 1400 survivors. Factor analysis identified eight factors related to the power to live: leadership, problem solving, altruism, stubbornness, etiquette, emotional regulation, self-transcendence, and active well-being. All factors had sufficient internal construct validity, and six of them showed significant associations with one or more measures of survival success in the disaster, including immediate tsunami evacuation, problem solving in refugee situations, recovery during reconstruction, physical health, and mental health. Overall, the personal characteristics described by the eight factors largely overlap with those described in previous arbitrary models. Further research should investigate the domains, phases, and contexts in which each factor contributes to survival, address whether the factors are rooted in nature or in nurture, and explore their psychological or physiological bases.

## Introduction

In disaster situations, people perceive, judge, and behave differently depending on their personal characteristics. When alerted by an alarm of an approaching tsunami, some people evacuate quickly, whereas others disregard the alarm. When faced with a shortage of food and commodities in refuge, some try to find ways to obtain these resources, whereas others simply endure. On the long path to reconstruction, some keep moving toward a better future, whereas others remain depressed and wait for help. There are likely many factors involved in such a “power to live” with disasters. Understanding the most important factors may contribute to systematic preparations for future disasters, by helping in the creation of education or training programs that aim to enhance people’s power to live, or by enabling the design of social systems that accommodate people who have different profiles with respect to this power. Indeed, the psychological analysis of the diverging tornado death rates between northern and southern states within the U.S. has provided an impressive demonstration of the importance of human characteristics for disaster mitigation [1]. Research has repeatedly pointed out that human factors can lead to bottlenecks that reduce the effectiveness of political or technological countermeasures against natural or technological disasters [2,3]. Similarly, the 2011 Great East Japan Earthquake disaster, in which a cutting-edge alarm system was not able to prevent the loss of 15,000 lives in a tsunami, re-emphasized the importance of human factors [4]. Moreover, abundant evidence is available with regard to the effects of personal characteristics on the mental health of people affected by disasters [5].

In addition to helping us survive infrequent disasters, the power to live may also help us to overcome difficulties in daily life. It may prevent us from becoming involved in crimes or in accidents. It may help us to overcome problems related our health, economic situations, and social relationships. In the long-term, it may have an impact on business, career development, and self-actualization. It is possible to conceive of disasters as falling at the extreme end of a range of life difficulties, uncertainties, or changes. This conceptual framework posits that the power to live captures aspects of an individual’s personality, ways of thinking, or customs in daily life, rather than being a special skill that is required only in exceptional situations. In other words, it underscores uncertainties, changes, and disasters as basic characteristics of our very daily life. Such a view can be found in the work of numerous experts [6–8].

For example, the American author Al Siebert questioned why some people manage to cope with difficult circumstances in life and eventually make things turn out well. He dedicated his life to interviewing survivors of various adversities in daily life, work, and health as well as survivors of more extreme situations, such as the World Trade Center attacks. He then extracted a set of essential personality factors of successful survivors that together comprise the “survivor personality” (SvP), [6]. Similarly, the German psychologist Paul B. Baltes dealt with the concept of “wisdom” as an intellectual virtue and as a means to live well despite the uncertainties of human life. Baltes’s attempt to bring this perennial philosophical concept into the laboratory was manifested in his Berlin Paradigm. In this paradigm, a broad definition of wisdom is combined with an expert knowledge system, which he called the “fundamental pragmatics of life” (FPL) [7]. As a further example, in 1996 the Japanese government renewed its educational policy by drawing attention to the rapidly changing nature of society. The term “zest for living” (ZfL) was chosen to describe the qualities and abilities that children need to thrive during periods of turbulent change. The new policies stressed the importance of fostering the right balance among the separate factors that contribute to zest for living [8].

These aforementioned advocates understandably, focused on different factors or domains as the major aspects of the power to live. Al Siebelt pointed to several essential factors of the SvP [6], arguing that playful curiosity, flexibility, a synergistic personality, empathy, and intuition are necessary for coping and thriving in a world of constant change. Furthermore, he suggested that a talent for serendipity, not being a “good” child, and having a strong sense of self (i.e., self-esteem, self-confidence, and self-concept) contribute to resilience in difficult situations. In Paul Baltes’s conceptual framework of wisdom, the FPL consisted of the following: rich factual and procedural knowledge, lifespan contextualism (which emphasizes that humans are products of their own decisions), value relativism, and the recognition and management of the fundamental uncertainty of life [7]. The FPL was assumed to apply to life planning (e.g., which future life goals to pursue and how), life management (e.g., how to deal best with critical problems), and life review (e.g., how best to make sense of our life history and past experiences). For the Japanese government, ZfL included the following: the ability to identify problem areas for oneself; the ability to learn, to think, and to make judgments; and the capacity to act independently and become more adept at problem solving. In addition, it was deemed necessary to imbue children with a rich sense of humanity in the sense that, while exercising self-control, they must be able to cooperate with others, have consideration for others’ needs, and possess a spirit capable of feeling emotion [8].

Despite the differences in the motivations, target populations, and approaches of these three models, it is noteworthy that there is considerable overlap among their proposed factors and domains. For example, the advantages of a strong capacity for problem solving, particularly when accompanied by a nuanced awareness of the context-dependence of solutions, are explicitly mentioned with regard to the FPL and ZfL. The problem-solving potential of the SvP is further decomposed into the qualities of a playful curiosity, a synergistic personality, and intuition. The importance of the “self” is also common across the three models. It is mentioned explicitly in the SvP, discussed in terms of life planning and review in the FPL, and appears implicitly in ZfL as integral to the characteristics of identifying problem areas for oneself and acting independently. Given the degree of overlap among the three models, it seems desirable to integrate them using an objective approach.

Thus far, empirical research regarding the personal characteristics that are advantageous for surviving disasters has been limited to studies of mental health, including post-traumatic stress disorder (PTSD), although a study of tornado evacuation provides one notable exception [1]. Examinations of personal characteristics have focused primarily on those personality traits or psychological resources that are related to the degree of post-traumatic distress experienced by survivors, as measured by previously established inventories. Higher levels of distress after a disaster have been related to higher neuroticism and a greater tendency for survivors to worry or experience anxiety. On the other hand, lower levels of distress have been related to higher levels of self-efficacy, mastery, self-esteem, hardiness, perceived control, a temporal orientation toward the future, optimism or hopefulness, and a sense of coherence [5]. Although mental health is, no doubt, important for survival in disasters and other adverse circumstances in our lives, it represents only a part of the power to live. For example, the characteristics that are essential for successful evacuation or for problem solving in refuge settings may differ from those that are important for mental health. A scientific exploration of the power to live that encompasses different aspects of survival in disasters and other adversities has yet to be performed.

Here, we attempt to objectively identify the major factors underlying the power to live by summarizing the responses of survivors of the 2011 Great East Japan Earthquake disaster. In previous research [9], we conducted personal interviews with 78 survivors regarding their survival experiences and elicited their opinions about the power to live that were relevant to those experiences. For the purpose of identifying the relationship between survival success and personal characteristics, and taking into account the context dependency of the relationship, extracted data from the interview were cross-tabulated and applied to a correspondence analysis. The results of this qualitative analysis demonstrated that different factors contribute to survival at different phases of the disaster, and indicated that relevant personal traits, attitudes, and habits act as classes of context-independent personal characteristics. In the current study, based on the previous results, we devised a questionnaire comprising 40 items pertaining to these three classes of personal characteristics. The battery also included a questionnaire related to survival success during disaster, such as the experiences of evacuees from the tsunami, the resolution of problems that arose in a refuge setting, and the subjective degree of the recovery of the state of the life, physical, and mental health. We obtained responses from > 1400 survivors; the majority of the respondents had evacuated to avoid the tsunami, had swept away or damages their residences, and/or had lost, or experienced a break from, their jobs. By performing a factor analysis on responses pertaining to personal characteristics, we created an inventory of major factors relevant to the power to live. To examine the external construct validity of the factors, we examined the relationship between factor scores, measures of survival success and certain demographic factors.

## Materials and Methods

### Ethics Statement

The protocols for both the interview and questionnaire surveys were reviewed and approved by the Ethics Committee for surveys and experiments of the Graduate School of Arts and Letters, Tohoku University (2012-1019-190749). The interviewees were initially advised of the purpose of the survey, with only those comfortable with the protocol subsequently participating. Consent was obtained by verbal means only, to reduce procedural burden, given that the respondents were disaster victims. The Ethics Committee approved our consenting procedure.

### Participants

Participants in both surveys were survivors of the 2011 Great East Japan Earthquake disaster, which occurred on March 11^th^, 2011. They were sampled from among the residents of Miyagi prefecture, where damage by the earthquake and tsunami was the most severe. We did not include people who, by chance, had vacated the severely affected region at the time of the earthquake.

Interviews were conducted between December 2012 and March 2013. Thirty-five males and 43 females, ranging in age from their 20s to their 80s, participated. Among them, 2.6% lost family members, 73.1% had lost their houses, and 19.2% had lost their jobs [9].

A questionnaire battery was sent to 3600 residents in the middle of December 2013. The residents were randomly sampled from the electoral register (i.e., 20 years of age or older) of tsunami-affected districts or temporary settlements in four cities: Kesen-numa (900), Ishinomaki (1800), Sendai (633), and Natori (267), which are the four most populated coastal cities in the Miyagi prefecture. A total of 1412 questionnaires (39.2%) were anonymously completed and returned by mid-January 2014. The respondents included 564 males and 832 females, most of whom ranged in age from their 20s to their 70s. Among them, 67.9% evacuated to avoid the tsunami of March 11th, 2011; 8.4% lost family members; 38.1% lost friends; 48.2% completely lost their residences; 40.3% suffered partial damage to their residences; 15.7% lost their jobs; and 41.8% experienced breaks from work. Their demographic profiles and disaster experiences are summarized in Table 1.

**Table 1:** Demographic profiles and disaster experiences of respondents.

Attribute		*N*	%
*Demographic characteristics*			
Age	20–29	87	6.2
	30–39	144	10.2
	40–49	216	15.3
	50–59	274	19.4
	60–69	391	27.7
	70–79	280	19.8
	80–	5	0.4
Sex	Male	564	39.9
	Female	832	58.9
*Disaster experience*			
Tsunami evacuation	Yes	959	67.9
Personal loss	Family members	119	8.4
	Friends	538	38.1
Residence	Total loss	681	48.2
	Partial damage	569	40.3
Job	Lost	221	15.7
	Break	590	41.8

### Construction of the questionnaire

In the interview, participants were asked to report three episodes in which they overcame or were currently overcoming crises or difficulties related to the 2011 Tohoku earthquake or tsunami; any episode occurring between the earthquake and the time of the interview was deemed relevant. For each episode, the participant was first asked to detail the episode itself and then to describe which of his or her personal characteristics might have contributed to any positive or successful aspects of the episode. To elicit the response, prompts such as the following were offered: “Not all the people in your situation could do that. Why could you do that? Is it possibly related to your personal characteristics?” The participants were encouraged to provide details about the characteristics they mentioned and were urged to be as specific as possible about their meaning and context, occasionally being asked to add other examples of daily or childhood behavior that reflected these characteristics [9].

We extracted phrases describing the participant’s “opinion” regarding the power to live from each transcript. Any phrases that could be interpreted as describing a personality trait, attitude, or daily habit that were deemed to be advantageous in the described episode or in situations similar to the episode were noted. A total of 718 such phrases were extracted from the first 78 interviews.

Next, the phrases were summarized and incorporated as questionnaire items following extensive discussion among the authors. Characteristics that were mentioned by three or more participants were retained. We excluded phrases that were so simple that they could have different meanings depending on the context. For example, we did not include “I am talkative,” but we did include “I take the initiative in talking to other people” or “Sophisticated words that move people’s hearts come out of my mouth.” Forty items were eventually constructed.

### Questionnaire survey

Participants used a six-point scale (0: not at all, 5: very much) to rate the degree to which each statement included in the questionnaire applied to themselves.

In addition to this personal characteristics questionnaire, the battery included a questionnaire related to the demographic characteristics and disaster experiences of the respondents. The latter included the following items related to survival success, which were then used to examine the external construct validity of the inventory under development:

#### Tsunami evacuation

Those who had evacuated the area in which they were situated when the major earthquake occurred (March 11^th^, 2011) having taken into consideration the risk of a subsequent tsunami, were asked whether they took this emergency evacuation action “immediately after the earthquake”. Immediate evacuation was considered successful.

#### Refugee-related problem solving

All participants were asked how they had managed the problems that arose in a refuge setting or in a comparable situation, The questionnaire focused on the following 18 essential daily issues: 1) eating, 2) cooking, 3) changing clothes, 4) room temperature, 5) sleeping, 6) toileting needs, 7) washing one’s face, 8) bathing, 9) washing clothes, 10) obtaining information, 11) transportation, 12) one’s own medical care, 13) the medical care of family members, 14) one’s own mental stability, 15) the mental stability of family members, 16) noise, 17) smell, and 18) privacy. Participants were first asked to rate if they had experienced any problems with each issue on a three-point rating scale (0: no, 1: a little, or 2: very much) and then to report whether they had solved the problems themselves. The ratio of the number of self-solved issues to the number of the issues experienced (i.e., 1 or 2) was considered the index of successful survival.

#### Reconstruction

All respondents were asked to subjectively evaluate the state of their lives at two time points: (a) just after the earthquake and tsunami, and (b) at the time of the survey. To summarize their impressions of the quality of their current daily lives in comparison with the state of their lives just before the earthquake, they were asked the following questions (a) “To what degree (%) has the state of your life deteriorated?” and (b) “To what degree (%) has the state of your life recovered?” We assessed the “recovery rate” of those whose lives had deteriorated by 50% or worse using the formula [(b)–(a) / 100 –(a)].

#### Physical health

All respondents were asked to rate (a) their physical condition immediately before the earthquake (March 11th, 2011), as well as (b) the current state of their physical condition using a six-point scale (0: healthy, 5: very bad). We reversed the value of the change in their physical condition as follows:-[(b)-(a)]; this was done so that positive values reflected improvement, with the expectation that there would be a negative average value due to the adverse effects of the disaster.

#### Mental health

The respondents’ mental conditions at the current time and prior to the disaster were assessed and analyzed in the same way as were their physical conditions.

### Analysis

A factor analysis was performed using a maximum likelihood method to determine the major factors of the power to live. Items that had a commonality of 0.3 or lower were eliminated. The number of factors for inclusion was determined using the Kaiser criteria and a scree plot. The promax rotation method was applied. Items with a loading of lower than .40 on all factors or items with loadings of .40 or higher on more than one factor (cross-loading) were eliminated. To evaluate the internal construct validity, Cronbach's *α* was calculated for each factor.

The relationships of the factor scores to age and sex were assessed using a one-way analysis of variance (ANOVA) and a two-sample *t*-test, respectively. Although the age data were originally obtained in the form of a seven-option choice (20s, 30s, 40s, 50s, 60s, 70s, or over 80), the over-80 group was merged with the 70s group due to the small sample size of the over-80 group.

To evaluate the external construct validity of each factor, we analyzed the relationship of that factor score to the degree of survival success based on the disaster-experience items. In terms of the item pertaining to tsunami evacuation, we compared the factor scores of those who evacuated immediately with those who did not (two-sample *t*-test). With regard to the other items (refugee-related problem solving, reconstruction, and physical and mental health), we computed the Spearman’s correlation coefficients between factor scores and the success indices. The statistical relationships are presented as follows: we describe as “significant” those relationships that meet the statistical criterion of *p* < .05 (two-tailed), corrected for multiple comparisons (i.e., number of factors) via the Bonferroni method; we describe as a “tendency” any relationship that meets the statistical criterion of *p* < .05 (two-tailed), but without correction.

## Results

In the factor analysis, five items were eliminated based on the commonality criteria, and one item was eliminated based on the loading criterion. Eight factors had eigenvalues greater than 1.00 (9.26, 2.55, 2.26, 1.69, 1.42, 1.17, 1.14, and 1.01) and accounted for 60.3% of the total variance. The scree plot showed an abrupt drop in loading between the eighth and ninth (0.84) factors. We therefore chose an eight-factor solution (Table 2): leadership (F1: five items), problem solving (F2: five items), altruism (F3: five items), stubbornness (F4: five items), etiquette (F5: three items), emotional regulation (F6: four items), self-transcendence (F7: four items), and active well-being (F8: three items). Cronbach's *α* exceeded .70 for all these factors.

**Table 2:** Factor analysis of the power to live.

	Items	F1	F2	F3	F4	F5	F6	F7	F8	α
**F1**	**Leadership**									.80
31.	To resolve problems, I gather together everyone involved to discuss the matter.	**.84**	.17	.01	-.01	-.13	-.11	-.08	.02	
40.	In everyday life, I often take the initiative to gather people together.	**.80**	-.06	.04	-.06	-.01	-.05	-.10	.19	
26.	I take the initiative in talking to other people.	**.57**	.03	.04	.03	.22	.00	-.02	-.12	
30.	Sophisticated words that move people’s hearts come out of my mouth.	**.51**	.12	.06	.02	.02	.01	.01	.03	
39.	In everyday life, I make sure to keep in contact with friends and acquaintances.	**.41**	-.11	-.03	-.04	.37	-.03	.03	.19	
**F2**	**Problem solving**									.78
24.	When I am fretting about what I should do, I compare several alternative actions.	.07	**.81**	-.13	.00	-.02	.00	-.02	.01	
25.	Before taking action, I think of a plan and the order of priority.	.01	**.69**	-.16	.05	.03	-.11	.14	.00	
33.	When talking to someone, I think about that person’s personality, wishes, and abilities and choose an appropriate attitude and words accordingly.	.02	**.55**	.08	-.12	.13	-.09	-.02	.08	
13.	The more agitated the people around me become, the calmer I somehow become.	.01	**.44**	.05	.13	.01	.25	-.15	-.08	
23.	To resolve a problem, I first of all initiate action.	.31	**.44**	.06	.04	-.05	.11	.07	-.07	
**F3**	**Altruism**									.77
5.	I like it when other people rely on me and are grateful to me.	.07	-.01	**.65**	.01	-.08	.03	-.03	.04	
1.	When I see someone having trouble, I have to help them.	.01	-.03	**.64**	-.01	.14	.06	.03	-.12	
6.	When someone asks me to do something for them, I cannot refuse.	-.17	-.05	**.63**	.04	.07	-.03	.03	.03	
4.	Other people’s good fortune makes me happy so I like to help others.	.17	-.10	**.58**	-.06	-.05	.06	.17	-.02	
2.	I am meddlesome and I like to do things for others.	.30	-.13	**.55**	.06	-.04	-.08	.00	-.06	
**F4**	**Stubbornness**									.75
8.	I am stubborn and always get my own way.	-.13	.05	.02	**.76**	.03	-.05	-.09	.04	
11.	I unhesitatingly say whatever it is I want to say.	.32	-.20	-.17	**.61**	-.05	.08	.09	-.12	
12.	I clearly distinguish between black and white: what’s good is good, and what’s bad is bad.	.12	-.03	-.14	**.58**	.09	.04	.17	-.07	
7.	I hate losing.	-.11	.07	.25	**.58**	.02	-.01	-.10	.04	
9.	I am highly motivated with regard to things that I like or want to do.	-.14	.14	.13	**.49**	-.07	-.09	-.01	.29	
**F5**	**Etiquette**									.71
38.	On a daily basis, I take the initiative in greeting family members and people living in the neighborhood.	.05	.03	.03	.02	**.80**	.02	-.07	-.01	
37.	In everyday life, I take care of myself as much as possible.	-.09	-.05	-.02	.04	**.60**	.09	.02	.09	
28.	When someone has helped me or been kind to me, I clearly convey my feelings of gratitude.	.02	.20	.08	-.02	**.54**	-.07	.04	-.03	
**F6**	**Emotional regulation**									.77
20.	During difficult times, I endeavor not to brood.	-.04	-.06	.03	.01	.02	**.96**	-.16	.05	
22.	During difficult times, I endeavor to think positively, telling myself that this experience will benefit me in the future.	.03	.13	.06	-.03	-.05	**.48**	.20	.05	
21.	During difficult times, I compare my circumstances with the situation around me and in society, and I think that matters cannot be helped.	-.20	-.03	-.03	-.03	.05	**.45**	.19	.09	
19.	When something happens, I try to stay calm and not panic.	.02	.37	-.06	-.05	.01	**.42**	.04	-.03	
**F7**	**Self-transcendence**									.76
17.	I am aware that I am alive, and have a sense of responsibility in living.	-.08	-.05	.01	-.02	-.04	-.02	**.84**	.05	
18.	I am aware of the path and teachings I should follow as a person.	-.12	.10	.05	.00	.11	.00	**.66**	.02	
15.	I am aware of the role I should play in society.	.18	.00	.06	.03	-.07	.04	**.45**	.07	
16.	I think that my actions toward others will go around and eventually come back to me.	-.08	.20	.15	.00	.01	-.07	**.44**	-.09	
**F8**	**Active well-being**									.74
34.	In everyday life, I have habitual practices that are essential for relieving stress or giving me a change of pace.	.03	.04	-.02	.01	.00	.08	-.01	**.64**	
36.	In everyday life, I have habitual practices that are essential for maintaining my physical health.	.14	-.11	-.11	.04	.14	.04	.07	**.57**	
35.	In everyday life, I endeavor to find opportunities to acquire new knowledge, skills, and attitudes.	.24	.17	.04	.00	-.09	.06	.01	**.46**	
	Final eigenvalues	6.20	6.31	4.56	3.31	3.34	5.24	5.82	3.94	

Factor loadings of .40 or higher are presented in bold typeface. α: Cronbach's α. Items were originally in Japanese and translated into English by the authors in consultation with a native English speaker.

The relationships of the factor scores to age, sex, and survival success are summarized in Table 3.

**Table 3:** Relationships of factor scores to age, sex, and survival success.

Attribute		*N*	F1	F2	F3	F4	F5	F6	F7	F8
	*Level*		*Factor score (Mean ± SD); F/t-value*							
Age	20–29	86	-.32 ± .92	-.05 ± 1.00	.14 ± .99	.08 ± .79	-.52 ± .91	-.46 ± 1.09	-.48 ± .93	.01 ± .94
	30–39	142	-.00 ± .84	.06 ± .77	.08 ± .89	.14 ± .73	-.20 ± .90	-.13 ± .89	-.13 ± .82	-.06 ± .80
	40–49	205	-.03 ± .98	.12 ± .87	.04 ± .82	.07 ± .85	-.12 ± .78	-.02 ± .96	-.03 ± .86	-.07 ± .83
	50–59	263	-.03 ± .90	-.02 ± .89	-.04 ± .86	-.11 ± .89	.01 ± .83	.00 ± .88	.03 ± .87	-.09 ± .84
	60–69	345	.05 ± .91	-.01 ± .91	.00 ± .87	-.08 ± .92	.18 ± .83	.08 ± .84	.13 ± .85	.06 ± .81
	70–	214	.14 ± 1.01	-.06 ± 1.08	-.08 ± 1.03	.07 ± 1.01	.16 ± .99	.19 ± .92	.09 ± 1.07	.13 ± .96
	*F(5*, *1249)*		3.26*	1.08	1.13	2.67*	12.86**	7.33**	7.44**	2.37*
Sex	Male	511	.04 ± .94	.10 ± .92	-.07 ± .92	.12 ± .87	-.32 ± .91	.05 ± .85	-.09 ± .88	.12 ± .82
	Female	743	-.02 ± .93	-.06 ± .92	.05 ± .89	-.08 ± .90	.22 ± .80	-.03 ± .96	.07 ± .92	-.07 ± .87
	*t(1252)*		1.03	3.06**	-2.47*	4.04**	-11.12**	1.55	-3.01**	3.90**
Tsunami evacuation (immediate)	Yes	449	.09 ± .97	.08 ± .97	.06 ± .95	.00 ± .92	.08 ± .88	.12 ± .95	.10 ± .93	.06 ± .89
	No	394	-.08 ± .89	-.06 ± .90	-.01 ± .87	-.05 ± .88	.02 ± .88	-.06 ± .86	.02 ± .87	-.04 ± .84
	*t(841)*		2.56*	2.19*	1.10	.82	.98	2.78**	1.40	1.72
	*Mean ± SD*		*Spearman’s correlation coefficient*							
Refuge-related problem solving (ratio solved) (%)	33.0 ± 30.5	1200	.053	.115**	.082**	.030	.051	.098**	.059*	.038
Reconstruction (recovery rate) (%)	68.0 ± 27.1	896	0.014	0.084*	-0.003	0.035	-0.028	0.042	0.043	0.076*
Physical health (improvement)	-.58 ± 1.43	1252	0.052	0.044	-0.020	0.077**	-0.059*	0.080**	0.008	0.108**
Mental health (improvement)	-.54 ± 1.57	1255	0.091**	0.072*	-0.014	0.051	-0.044	0.120**	0.046	0.147**

For age, sex, and tsunami evacuation, the sample size (*N*) and factor score (mean ± *SD*) for each factor are shown for each level. Statistical values are *F*-values for age (ANOVA) and *t*-values for sex and tsunami evacuation (two-sample *t*-test). For refugee-related problem solving (% solved by oneself), reconstruction (% recovery), physical health (improvement on a six-point scale), and mental health (improvement on a six-point scale), the factor score (mean ± *SD*), sample size (*N*), and Spearman’s correlation coefficient for each factor are shown.

*: *p* < .05

**: *p* < .05, corrected for multiple comparisons (i.e., the number of factors) using the Bonferroni method.

The association of the factor score with age was significant for F5, F6, and F7, and it reached the level of a tendency for F1, F4, and F8. A *post hoc* comparison across age groups was corrected to *p* <.05 for multiple comparisons using the Tukey method. For F1, the score was significantly higher among those in their 60s and those 70 or older than among those in their 20s. For F5, the score was significantly higher in all age groups over 40 than in those in their 20s, and it was higher in those in their 60s and in those 70 or older than in those in their 30s and 40s. For F6, the score was significantly higher in any age group over 40 than in those in their 20s, and it was higher in those 70 and older than in those in their 30s. For F7, scores were significantly higher in among those 30 and older than in those in their 20s.

In terms of sex differences, scores were significantly higher among males for F2, F4, and F8, and they were significantly among females for F5, F7, with the same tendency for F3.

The scores of the various factors revealed significant associations with survival success, with one or more significant associations found for each disaster item. With regard to tsunami evacuation, a higher score for F6 was significantly associated with immediate evacuation when the earthquake occurred, and factors F1 and F2 showed the same tendency. In terms of refugee problem solving, higher scores for F2, F3, and F6 were significantly associated with successful individual problem solving in refugee situations, with F7 showing the same tendency. In terms of reconstruction, higher scores for F2 and F8 showed a tendency toward an association with a better recovery rate. With regard to physical health, higher scores for F4, F6, and F8 were significantly associated with little or no deterioration in one’s current physical condition compared with one’s condition prior to the earthquake; however, scores for F5 showed the inverse tendency. In terms of mental health, higher scores for F1, F6, and F8 were significantly associated with little or no deterioration in one’s current mental condition compared with that prior to the earthquake, and F2 showed the same tendency.

## Discussion

By summarizing the opinions of survivors of the 2011 Great East Japan Earthquake disaster, we identified eight major factors that correspond to the power to live: leadership (F1), problem solving (F2), altruism (F3), stubbornness (F4), etiquette (F5), emotional regulation (F6), self-transcendence (F7), and active well-being (F8). All these factors had sufficient internal construct validity in terms of Cronbach's *α*. Six of them (i.e., except F5 and F7) showed a significant association with at least one measure of survival success in the disaster, further indicating their external construct validity. Their association with different survival measures is consistent with the notion that each factor contributes to different aspects of survival at different phases of the disaster.

Here, we compare each factor with the existing models that address the power to live (i.e., SvP, FPL, and ZfL):

Leadership (F1) represents the attitude or habit of gathering and organizing people. Although this factor has not been explicitly identified in previous models, it appears to be an embodiment of previously proposed factors, such as the “synergistic personality” in SvP and the ability to “cooperate with others” in ZfL; leadership habits or skills are likely to play a major role in the effectiveness of these previously identified factors. The factor score for leadership was higher for people in their 60s than for people in their 20s, probably reflecting the differential amounts of social experience of these age groups. A significant positive association of this factor with mental health was observed. Whereas this association is not straightforward, it is consistent with the notion of “positive human health” [10,11], which encompasses both physical and social/psychological functioning. A potentially related personality trait is self-compassion, which has been shown to be associated with psychological functioning related to leadership (e.g., optimism, self-initiative) as well as with mental health [12,13].

Problem solving (F2) represents the attitude or habit of strategically tackling problems. This factor is one of the major targets of all previous models. In particular, the concept overlaps well with the important capacity in ZfL: “To identify problem areas for oneself, to learn, think, make judgments and act independently, and to become more adept at problem solving.” In the FPL this skill appears to be a major component of the expert knowledge relevant to life planning and management. In the SvP, the factor appears to be broken down into three domains: cognitive characteristics (i.e., intuition), behavioral characteristics (i.e., a synergistic personality), and a personality that promotes skill acquisition (i.e., playful curiosity). The score for this factor was significantly higher for males than for females, which is consistent with the male inclination toward a “systemizing” cognitive style (i.e., a tendency to analyze the variables and underlying rules of a system and to predict and control its behavior) [14]. As expected, this factor was advantageous during all phases of the disaster. A significant positive association was observed between this factor and successful problem solving in refugee situations. Similar tendencies were also identified with regard to immediate tsunami evacuation, recovery rate during reconstruction, and mental health.

Altruism (F3) represents the personality trait that causes people to care about and help others. This factor aligns with the emphasis in ZfL on “a rich sense of humanity” that drives people to consider the needs of others. It is also partially included in the notion of empathy, as discussed with regard to the SvP. According to our data, the score for this factor tended to be higher among females than males, which is consistent with the female inclination toward an empathic cognitive style [14]. Interestingly, this factor showed a significant positive association with problem solving in refugee situations, which addresses the survival of the self, a fact that is seemingly inconsistent with the literal meaning of altruism. However, there are two possible ways of reconciling this apparent contradiction: First, one’s altruistic behavior may contribute to one’s own survival through group dynamics, perhaps by involving reciprocal altruism or the enhanced survival of the group [15]. A second and contrasting interpretation is that one can afford to be altruistic only when one has resources to spare.

Stubbornness (F4) represents the personality trait, attitude, or habit of sticking to one’s desires or beliefs. This factor is consonant with the resilient personality included in the SvP of “not being a good child” and “strong self-confidence”, which appear to cover the behavioral and motivational aspects, respectively, of this factor. It is also consonant with the FPL notion of having clarity about one’s future life goal and with the ZfL capacity “to learn, think, make judgments and act independently” and with having “a spirit that feels emotion”. The factor score was significantly higher for males than females, and was positively associated with physical health. These relationships appear consistent with empirical data [16,17].

Etiquette (F5) represents the attitude or habit of conforming to social norms in daily behavior. This factor may be advantageous for maintaining good relationships with family and community members. Although none of the previous models have explicitly identified this factor, it seems related to procedural or strategic knowledge included in the FPL and to the importance of exercising self-control and cooperating with others in ZfL. The factor score was higher among the elderly than the young and higher among females than males. However, the score was associated with none of the survival measures analyzed in the current study; there was even a tendency for a negative association with physical health, potentially reflecting its positive association with age.

Emotional regulation (F6) represents the attitude or habit of endeavoring to stay calm in difficult or strained circumstances. This factor comprises the “serendipity talent” of the SvP and coincides with “exercising self-control” in ZfL. Scores on this factor increased significantly with age, and this factor was significantly positively associated with immediate tsunami evacuation, problem solving in refugee situations, physical health, and mental health. Its particularly strong contribution to mental health is in good agreement with the previously noted relationship between post-disaster distress and high degrees of neuroticism and tendencies to worry or experience anxiety [5].

Self-transcendence (F7) represents awareness of the meaning of one’s life from a spiritual perspective. This factor seems to exemplify the strong self-concept in the SvP, and to be relevant to the FPL notions of life planning and life review. This factor is likely to drive one to cooperate with others, a behavior that is emphasized in ZfL. Scores on this factor were higher among elderly than among young individuals and higher among females than among males. This factor tended toward a positive association with problem solving in refugee situation. It is worth considering whether this factor has a particular causal relationship with disaster experiences, given its overlap with the positive psychological outcomes reported by people who have experienced traumatic events [18]. Specifically, the “appreciation of life” factor in the Posttraumatic Growth Inventory (PTGI) aligns well with this factor.

Active well-being (F8) refers to the daily practice of maintaining or improving one’s physical, mental, and intellectual status. This factor has rarely been addressed in previous models although such a practice is likely to be consequential. Its omission may be due to the fact that the notion of a “practice” is outside the scope of previous models or because the factor is not characteristic of survival in the face of changes or difficulties more generally. We know that it is not easy to maintain such a daily practice over a long period of time. A strong self-concept (in the SvP), skills for managing life’s uncertainties (in the FPL), or the ability to act independently (in ZfL) may aid in successfully maintaining active well-being. Scores on this factor were significantly higher among males than females. Its significant positive associations with physical and mental health are not surprising, as this factor was defined as the set of practices that enhance well-being. A tendency for a positive association with recovery during reconstruction appears noteworthy, given that this factor may provide an extraordinarily simple answer to the question of what should be done to enhance reconstruction after a disaster.

Overall, a systematic comparison of the current results with previous models reveals that the personal characteristics described by the eight factors relevant to the power to live largely overlap with those described in the previous models (SvP, FPL, and ZfL).

The most obvious advantages of the current model involve its objectivity with regard to sorting through relevant factors and its inventory for an empirical evaluation of the model. Previous models were subject to biases in the life values, preferences, or experiences of those who developed them, and they did not have a measurement tool; on the other hand, these models offered advantages in terms of their philosophical depth and systematic structure. We do not mean to suggest that the current model is free of bias. Indeed, the model is potentially biased by the common experience of its participants, the 2011 Great East Japan Earthquake disaster. The results could have been more or less different if the model had been constructed based on the opinions of survivors of other types of disasters or life difficulties. In addition, the technical details we chose may have introduced a source of bias, including the methods involved in interviewing or in summarizing the respondents’ opinions, the method of sampling participants, and the specific statistical methods we used. Depending on the intended purpose for the model, it should be critically validated and optimized; this is possible using the inventory.

The contribution of each factor to survival success is far from conclusive. First, the data on survival success may have been affected by deterioration of, and biases in, responders’ memories. There were intervals of 2 and 3 years between the disaster and the interview, and the disaster and the questionnaire survey, respectively. It is of note that two items loading onto F6 (i.e., items 21 and 22) appear to suggest a survival advantage is conferred by some form of memory bias. Second, we did not collect objective medical information pertaining, for example, to diagnosis (e.g., post-traumatic stress disorder, anxiety disorder, depression), health indices (e.g., blood pressure) and treatment (e.g., medication, psychotherapy), which may have exerted a significant impact on mental health and other subjective evaluations. Finally, numerous domains, phases, and contexts, to which each factor may contribute, remain to be examined in future. In particular, the idea of survival success through group dynamics has not been explored in the current research. Several factors, such as F1, F3, F5, and F7 may be more advantageous to the survival of groups than of individuals.

The issue of whether each factor has its origins in nature or in nurture is critical, particularly for the purpose of using this model to foster the power to live. If a factor can be strengthened by experience, it is a good target for education and training. On the other hand, if it is innate, we should abandon attempts to enhance it and, instead, should try to find a way to compensate for individual differences. With respect to the nature/nurture question, the descriptive form of the items does not offer much help. Some factors are defined in terms of personality traits, whereas others are defined in terms of attitudes or habits. However, the causal connections are not clear; it is possible that some habits are driven by certain personality traits, or that some habits foster a certain personality. As far as the current data are concerned, associations with age may be of interest. The factors with scores that increase with age (i.e., F5, F6, and F7) may reflect the benefits of experience. In contrast, age-independent factors (i.e., F2 and F3) may be innate.

The psychological and physiological bases of each factor are also of tremendous academic interest. It would be worth exploring the association of each factor with known personality traits, intellectual, affective, or procedural abilities, and patterns of neural processing. The findings would be relevant to the nature/nurture issue and to questions regarding the factor’s contribution to survival success. Furthermore, the current model, as well as similar models that deal with human characteristics related to survival success, may provide a critical link between basic personality and cognitive science research and successful human adaptation in the real world.

## Conclusion

We identified eight major factors that are central to the power to live in the face of disaster. To this end, we summarized the opinions of survivors of the 2011 Great East Japan Earthquake disaster. The eight factors are: 1) leadership (the attitude or habit of gathering and organizing people), 2) problem solving (the attitude or habit of strategically tackling problems), 3) altruism (the personality trait that leads people to care about and help others), 4) stubbornness (the personality trait, attitude, or habit of sticking to one’s desires or beliefs), 5) etiquette (the attitude or habit of conforming to social norms in daily behavior), 6) emotional regulation (the attitude or habit of endeavoring to stay calm in difficult or strained circumstances), 7) self-transcendence (the awareness of the meaning of one’s life from a spiritual perspective), and 8) active well-being (the daily practice of maintaining or improving one’s physical, mental, and intellectual conditions). All factors had sufficient internal construct validity, and six of them showed a significant association with at least one measure of successfully surviving this disaster. Overall, the personal characteristics described by the eight factors largely overlap with those described in the previous models. The advantages of the current model include its objectivity in identifying the factors and its empirically assessable inventory. Future research should focus on detailed investigations of the domains, phases, and contexts in which each factor contributes to survival, on their psychological or physiological bases, and on the question of nature versus nurture.

## Supporting Information

S1 File Raw questionnaire data set. (XLSX)
